# Monocular visual deprivation in Macaque monkeys: A profile in the gene expression of lateral geniculate nucleus by laser capture microdissection

**Published:** 2008-08-04

**Authors:** Georgiana Cheng, Henry J. Kaminski, Bendi Gong, Lan Zhou, Denise Hatala, Scott J. Howell, Xiaohua Zhou, Michael J. Mustari

**Affiliations:** 1 Department of Pathobiology, Cleveland Clinic, Cleveland, OH; 2 Department of Neurology, Case Western Reserve University, Cleveland, OH; 3 Department of Neurology and Psychiatry, Saint Louis University, St. Louis, MO; 4 Department of Neurology, Cleveland Clinic, Cleveland, OH; 5 Department of Dermatology, Case Western Reserve University, Cleveland, OH; 6 Visual Sciences Research Center, Case Western Reserve University, Cleveland, OH; 7 Ireland Comprehensive Cancer Center, Case Western Reserve University, Cleveland, OH; 8 Yerkes National Primate Research Center and Department of Neurology, Emory University, Atlanta, GA

## Abstract

**Purpose:**

Amblyopia is the most common cause of visual impairment in children. Early detection of amblyopia and subsequent intervention are vital in preventing visual loss. Understanding the molecular pathogenesis of amblyopia would greatly facilitate development of therapeutic interventions. An animal model of amblyopia induced by monocular vision deprivation has been extensively studied in terms of anatomic and physiologic alterations that affect visual pathways. However, the molecular events underlying these changes are poorly understood. This study aimed to characterize changes of gene expression profiles in the lateral geniculate nucleus (LGN) associated with amblyopia induced by monocular visual deprivation.

**Methods:**

Monocular vision deprivation was generated by either opaque dark contact lens or tarsorrhaphy of newborn rhesus monkeys. LGN was harvested at two or four months following induction of vision deprivation. Laser capture microdissection was used to obtain individual LGN layers for total RNA isolation. Linear T7-based in vitro RNA amplification was used to obtain sufficient RNA to conduct DNA microarray studies. The resulting Affymetrix GeneChip Expression data were analyzed using Affymetrix GeneChip Operating Software. Real-time quantitative polymerase chain reaction and in situ hybridization were used to further analyze expression of selected genes.

**Results:**

Using 52,699 microarray probe sets from a Rhesus array, we identified 116 transcripts differentially expressed between deprived and nondeprived parvocellular layers: 45 genes were downregulated and 71 genes were upregulated in deprived parvocellular layers. We also observed substantial changes in deprived magnocellular laminae: 74 transcripts exhibited altered expression, 42 genes were downregulated, and 32 genes were upregulated. The genes identified in this study are involved in many diverse processes, including binding (calcium ion binding, nucleic acid binding, and nucleotide binding), catalytic activity, and signal transducer activity.

**Conclusions:**

There were significant differences in gene expression profiles between deprived and nondeprived parvocellular layers and magnocellular laminae of LGN. These alterations in gene expression may play a critical role in the molecular pathogenesis of amblyopia. The genes identified in this study may provide potential targets for therapeutic intervention of this disease.

## Introduction

Amblyopia is the most common and significant disorder of spatial vision in children. It is characterized by a constellation of progressive visual deficits, including deficiencies in spatial vision, contrast sensitivity, grating acuity, and flicker sensitivity. These vision deficits, in turn, severely impair eye alignment and eye movement control. The severity of amblyopia depends on the magnitude, age of onset, and duration of visual deprivation. It can produce severe acuity loss, even leading to functional blindness in an eye, if not appropriately identified and treated.

To prevent amblyopia, adequate visual sensory experiences must be maintained throughout postnatal development. The postnatal window, when the visual system is developing, is referred to as the critical period. During this critical period, adequate visual sensory experience must be maintained to allow for the proper development of the visual system, especially in the lateral geniculate nucleus (LGN) and primary visual cortex (V1). Aberrant visual experience (e.g., unilateral cataract) during the critical period produces irreversible functional alterations in LGN and V1, ultimately leading to amblyopia.

Our current understanding of the etiology and pathogenesis of amblyopia comes largely from clinical observations and electrophysiological studies in children and from anatomic/physiologic studies in nonhuman primate models. Although these studies have added to our understanding of amblyopia, little is known about what causes amblyopia at the molecular level. Important questions yet to be answered include the following: Are gene and protein expression patterns altered at different levels of visuomotor relay and processing pathways? What cellular-level signaling pathways are responsible for the physiologic changes that produce amblyopia? This study attempts to address these relevant questions.

We hypothesized that the anatomic and physiologic changes associated with amblyopia are regulated by genes and proteins, and characterization of changes in gene expression profiling would allow identification of important genes involved in the molecular pathogenesis of amblyopia. To address this hypothesis, we used rhesus macaque monkeys as our model system to study gene expression profiles for the following reasons: First, humans and rhesus monkeys have similar visual-oculomotor behavior and primary visual pathways. Other animals such as rodents, rabbits, cats, and even New World primates do not have the ability to perform volitional smooth-pursuit. Second, the visual systems and the structure of LGN of humans and rhesus monkeys are similar in terms of organization and susceptibility to early problems in visual experience. Third, rhesus monkeys have already been accepted and used as an animal model for studying visual cortical function and performance. Therefore, rhesus monkeys provide unique advantages for gene expression profiling studies of amblyopia.

Monkeys with monocular vision deprivation are one of the most accepted and validated models for amblyopia [1,2]. The monkey visual system exhibits two distinct critical periods: birth to eight weeks and eight weeks to one year [3]. During the first critical period, monocular vision deprivation initially produces transient hypertrophy of neurons in the nondeprived parvocellular laminae. During the second critical period, the size of the neurons of the nondeprived parvocellular laminae is restored and the neurons of the deprived parvocellular laminae undergo atrophy [4-7]. The atrophic phase is much less pronounced in the magnocellular layers of both anisometropic and strabismic amblyopes [8,9]. Both magnocellular and parvocellular pathway are affected by monocular vision deprivation or amblyopia. Recent studies suggest that koniocellular (K) LGN cells (intercalated between the laminae) make up the koniocellular pathway [10-13]. Recent studies also suggest that LGN neuron size alterations are a consequence of interactions between V1 and LGN, but little is known about the cellular and molecular events behind these changes [3,14,15].

In the LGN of the monocular vision-deprived monkey, three layers are deprived and three other layers are nondeprived. Taking the whole LGN for study of the gene expression profile would not allow us to compare and contrast gene expression in deprived and nondeprived layers. Therefore, we used laser capture microdissection (LCM) to obtain individual deprived or nondeprived layers. These layers were then coupled with the Rhesus Macaque Genome Array to compare gene expression patterns between deprived and nondeprived layers of monocular vision-deprived LGN. We identified several genes with altered expression patterns, which might play an important role in the molecular pathogenesis of amblyopia.

## Methods

### Animals and tissue preparation

Seven monkeys were used in our study. Infant monkeys were born in captivity at Yerkes National Primate Research Center (YNPRC), Atlanta, GA. The infants were hand raised in a dedicated infant monkey nursery facility at YNPRC. Infants were fed standard infant formula and diet on an approved feeding schedule according to age and size. No food or water restrictions were used in association with any of our testing. Two normal control rhesus monkeys (*Macaca mulatta*), designated C1 and C2, were reared under a normal 12 h:12 h light-dark cycle for four months. Two rhesus monkeys, designated MD1 and MD2, were monocular vision-deprived by placing an opaque, dark contact lens, in each animal’s left eye. The uniocular contact lens rearing began at birth and extended for two months. The extended wear, gas permeable lens was replaced on a daily basis with a like sterilized opaque contact lens. Three rhesus monkeys, designated MD3, MD4, and MD5, were vision-deprived in the left eye at birth by tarsorrhaphy and were observed for four months. Testing visual function of the deprived eye occurred at 4 months of age after the eyelid of the deprived eye was opened. All monocular vision-deprived monkeys were reared under the same conditions as normal control monkeys. Following deep sedation with Telazol (4 mg/kg, I.M.), monkeys were sacrificed by delivering a bolus of Nembutol (90 mg/kg, I.V.). Both left and right LGN were dissected out, immediately embedded in optimal cutting temperature (OCT) compound (Sakura Finetek, Torrance, CA), snap frozen in liquid nitrogen, and kept at −80 °C before cryostat sectioning. The LGNs of monkeys MD1 and MD3 were used for LCM and microarray gene expression studies. All animal procedures were approved by the Institutional Animal Care and Use Committee at Emory University and Case Western Reserve University.

### Acuity testing in infant monkeys

We used a two-alternative, forced-choice, preferential looking (FPL) method to assess the visual acuity of our monkeys during monocular viewing conditions. Visual acuity of each eye was assessed during the first two months or four months by precisely calibrated Teller Acuity Cards using the general procedures outlined in the acuity card manual (Vistech Consultants, Inc., 1990, Dayton, OH). Each card had black and white stripes of a particular spatial frequency on one side, surrounded by an isoluminant gray background. Briefly, two experimenters referred to as the “observer” and the “holder,” participated in testing each monkey. The holder positioned the infant monkey in front of a gray screen that has an open window at its center. The observer sat on the other side of the screen and placed a test card in the window where the monkey could view it. For monocular vision testing, a small circular patch or contact lens occluder was placed over the fellow eye. This procedure was repeated using cards with various spatial frequencies until the highest spatial frequency that the monkey could locate was determined. We carefully monitored the disposition of the infant during this procedure to be certain that it was relaxed and attentive.

Infant monkeys naturally prefer looking at a patterned stimulus (grating) compared to a uniform gray level field. When these stimuli were presented, the infant looked toward the grating, provided its spatial frequency was not too high. The infant’s looking behavior fell to chance performance as the spatial frequency of the grating was increased. Therefore, FPL provides a well established clinical tool for use in noncooperative subjects (e.g., infant monkeys) to assess normal and abnormal acuity development [16]. Our monocular vision-deprived monkeys were able to reliably look at gratings of up to 19 cycles/degree using the eye that had unaltered vision during the rearing period. In contrast, no reliable preferential looking was observed when testing the deprived eyes of our animals, even for low spatial frequency stimuli (<1.0 cycles/degree). These findings were consistent with profound amblyopia of the deprived eye.

### Laser capture microdissection

**Figure 1:**
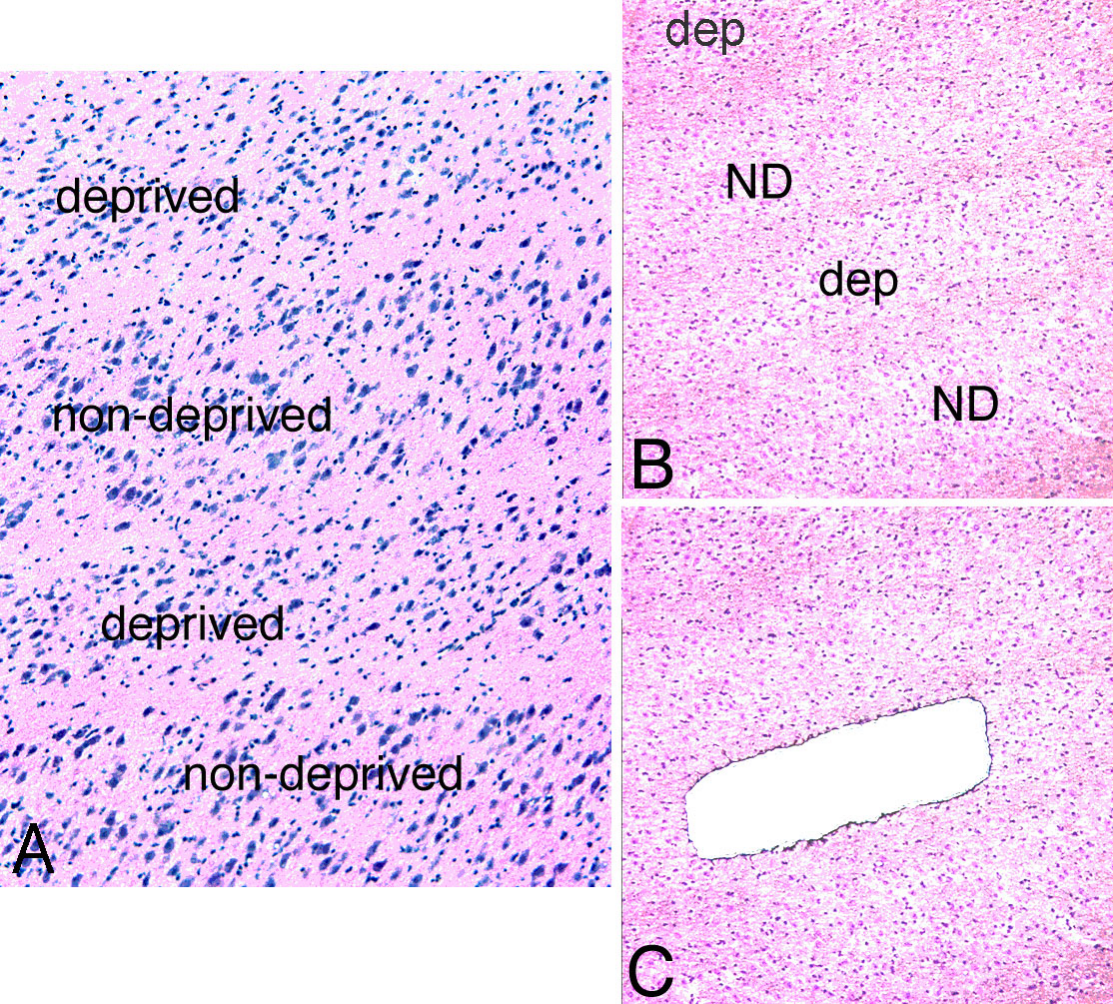
Morphology of LGN parvocellular layers from a monkey with monocular vision deprivation. **A:** H&E staining of lateral geniculate nucleus (LGN) sections showed neuronal shrinkage in deprived parvocellular layers as compared with non-deprived layers. **B:** Low power magnification deprived layer of LGN sections before laser capture microdissection (LCM). **C:** Low power magnification deprived layer of LGN sections after LCM. Abbreviations: dep: deprived layer; ND: non-deprived layer.

OCT-embedded LGN tissue was sectioned at 10 μm using a cryostat and placed on Leica membrane slides (PET-Membrane, Wetzlar, Germany). The slides were immediately transferred to a −80 °C freezer, LGNs of MD1 and MD3 monocular vision-deprived monkeys were used for the LCM and microarray study. Rapid toluidine blue staining was used to identify different layers. Briefly, the sections were fixed in 75% ethanol for 30 s, and then immersed in 0.05% toluidine blue in 20% ethanol for 30 s, followed by dehydration and wash with graded alcohols (75%, 95%, and 100%) for 20 s each. Four parvocellular layers and two magnocellular layers from each LGN were subjected to laser capture microdissection. Six layers represented nondeprived layers (layers I, IV, and VI of ipsilateral, layers II, III, and V of contralateral deprived eye) and six layers of LGN represented monocular vision-deprived layers (layers II, III, and V of ipsilateral, layers I, IV, and VI of contralateral). The Leica Biosystems AS LCM was used for laser capture microdissection. Each parvocellular and magnocellular layers were microdissected by LCM to exclude the koniocellular layer, as shown by Figure 1. These different layers were placed into the RNA sample buffer [17] immediately after dissection.

### RNA isolation, linear amplification and hybridization for microarray

We pooled LCM samples from 6–10 sections of each individual layer from each LGN. Total RNA from each layer was isolated using the PicoPure RNA isolation kit with DNase I digestion (Arcturus, Mountain View, CA). The quality of isolated RNA from LCM samples was measured by NanoDrop, Wilmington, DE. A260/A280 ratios were between 1.9 and 2.2 for all LCM samples. Two rounds of linear T7-based RNA amplification were then performed. The protocol of RNA amplification was generously provided by Dr. Eric Hoffman (Children's National Medical Center, George Washington University, Washington, DC). Similar techniques have previously been conducted in our laboratory [17] and others [18-20]. RNA amplification allowed for the production of microgram quantities of antisense RNA (aRNA) without distorting gene-to-gene ratios to obtain sufficient RNA for DNA microarray studies, as previously described [17,21,22]. Briefly, we used 100–250 ng of total RNA as starting material and applied two rounds of RNA amplification in which first-strand synthesis yielded cDNA incorporating a T7 promoter primer, and second-strand synthesis yielded double-stranded cDNA (cDNA synthesis kit from Invitrogen, Carlsbad, CA). Following cDNA purification, in vitro transcription (IVT) using T7 RNA polymerase yielded antisense RNA (cRNA; MEGAscript kit; Ambion, Austin, TX). Then, cRNA was used in the second round of amplification for first-strand cDNA synthesis with random primers and second-strand cDNA synthesis with T7 promoter primer. This yielded double-stranded cDNA, which was used in a second IVT reaction to generate biotinylated cRNA using T7 RNA polymerase and biotin-labeled nucleotides (Affymetrix, Santa Clara, CA). Biotin-labeled cRNA was used for hybridization according to the directions in the Affymetrix GeneChip Expression Analysis Manual. Briefly, after purification and fragmentation, 15 μg cRNA was added in a 300 μl hybridization mixture containing spiked IVT controls. Approximately 200 μl of this mixture was hybridized to DNA microarrays chip for 16 h at 45 °C in an Affymetrix GeneChip Fluidics Station 400. A Hewlett-Packard Gene Array Scanner was used for microarray scanning.

### Microarray data analysis

Affymetrix GeneChip Operating Software (GCOS) version 1.4 was used for initial data processing and fold ratio analysis. The GCOS algorithm analyzes data by 1) assessing probe pairs for perfect match and mismatch to determine the presence or absence calls of individual genes; 2) normalizing raw values to calculate their relative expression levels; and 3) employing statistical techniques to make increase, decrease, or no change calls. Any transcripts with expression intensities below 100 across all the samples were excluded since distortion of the *n*-fold difference values results when expression levels are low and may be within the level of background noise (background noise averaged 54 for 24 samples). Transcripts that had an absent call in all the samples were also eliminated from further analysis.

Pairwise comparisons were used between deprived layers and nondeprived layers of parvocellular or magnocellular laminae in the same LGN of the monocular vision-deprived monkey. For comparisons of deprived and nondeprived parvocellular layers, transcripts defined as differentially regulated met the following criteria: six out of eight increase/decrease calls in deprived layers versus nondeprived layers in eight replicates from the parvocellular layers; and absolute value of the average fold difference value ≥ 1.7. For deprived and nondeprived magnocellular layers, transcripts were defined as differentially regulated if they met the following criteria: four out of four increase/decrease calls in four replicates; and absolute value of the average fold difference value ≥ 1.7.

The quality of the microarray results were evaluated by comparing results of genes that were represented in the Affymetrix chip more than once [23,24]. To determine the function of genes, we used GeneSpring software GX 7.3.1 (Agilent Technologies, Foster City, CA) and methodology of GeneSpring in conjunction with resources provided by Weizmann Institute of Science GeneCards online databases and the National Center for Biotechnology Information (NCBI) Entrez.

### Real-time quantitative polymerase chain reaction technique

Selected transcripts were verified by qRT–PCR in triplicate. Briefly, transcript-specific primers were generated based on human sequences from GeneBank and were designed using Primer Express software (Applied Biosystems, Inc., Foster City, CA). NCBI BLAST was used to ensure specificity of each primer pair for each transcript. Reverse transcription and qRT–PCR was performed with an ABI 7000 sequence detection instrument. Monkey glyceraldehyde phosphate dehydrogenase (GAPDH) was used as an internal control to normalize the qRT–PCR data. Mean fold changes between the two study groups were calculated by averaging the triplicate measurements for each gene. The relative fold difference calculation used the 2^-∆∆CT^ method [25].

### In situ hybridization

The PCR primers were designed based on human gene sequences derived from GenBank. PCR reactions were performed using monkey LGN for corticotrophin-releasing hormone (CRH) and gamma-aminobutyric acid A receptor, alpha 1 (GABRA1).

The PCR products were subcloned into a pCR II vector using the TA Cloning Kit (Invitrogen), and each sequence was confirmed by sequencing (CRH monkey in situ probe sequence has 97% homologies with its human sequence, and GABRA1 monkey in situ probe sequence has 98% homologies with its human sequence). Digoxigenin (DIG)-labeled cRNA probes were made using the DIG RNA labeling kit (SP6/T7) and the protocol provided by Roche. The labeled probes were precipitated with ethanol, and then washed to remove unincorporated digoxigenin-labeled nucleotides. Tissue blocks containing the LGN were sectioned at 10 µm on a cryostat. Nonradioactively labeled probe in situ hybridization procedures were performed using the method described by us previously [17,26]. Briefly, the slides were dried for 2 h, fixed in 4% paraformaldehyde/PBS for 30 min at room temperature, washed with PBS, treated with Proteinase K at 1 µg/µl for 15 min at room temperature, acetylated for 10 min and then prehybridization was performed at room temperature for 2 h. The DIG-cRNA probe was heated at 80 °C for 5 min and chilled on ice. Hybridization was performed at 65 °C overnight in a humidified chamber in hybridization buffer that consisted of 50% formamide, 5X SSC, 5X Denhardts, 250 µg/ml baker’s yeast RNA, and 500 µg/ml salmon sperm DNA. The slides were washed with 0.2X SSC at 65 °C for 3 h. They were then blocked with 10% heat-inactivated sheep serum for 1 h followed by incubation overnight in an anti-DIG antibody (anti-DIG-AP; Fab fragment, Roche, Nutley, NJ) that was diluted 1:5000 in buffer consisting of 100 mM Tris pH 7.5, 0.15 M NaCl. Visualization of the signal was detected with 4-nitro blue tetrazolium chloride/5-bromo-4-chloro-3-indolyl-phosphate (NBT/BCIP), and the alkaline phosphatase reaction was stopped by washing with Tris EDTA buffer (10 mM Tris pH 7.5, 1 mM EDTA). The slides were then dehydrated and mounted with permanent mounting medium.

### In situ hybridization quantification

Images were collected with an RT Slider digital camera (Diagnostic Instruments, Sterling Heights, MI) mounted on an Olympus BX 60 Microscope using a 2X objective. Resulting 12 bit monochrome images were analyzed using Metamorph software (Universal Imaging, Downington, PA). Briefly, individual regions were drawn to differentiate the six layers of monkey LGN. Within each region the amount of positive in situ signal was selected by thresholding. The percent thresholded represents the percentage area of positive in situ signal contained in a given region. All images were collected, calibrated and analyzed using identical parameters. Image data were then exported to Excel for further analysis and signal quantification.

## Results

### Generating amblyopic monkey model

The deprived monkeys showed visual acuity in the viewing eye similar to that of normal age-matched control monkeys. In contrast, the vision-deprived eye, tested at two months of age for two monkeys and four months of age for three monkeys with the FPL method, had no measurable acuity even when the grating stimuli were less than 1 cycle/degree.

### Altered gene expression in lateral geniculate nucleus-deprived laminae of monocular vision-deprived monkey

All 24 samples (12 samples were from LGNs of monkeys that were monocular vision-deprived for two months and 12 samples were from LGNs of monkeys that were monocular vision-deprived for four months) were hybridized to the Rhesus Macaque Genome Array. The percentage of genes on the microarray reported as present in 24 samples ranged 43.7%–52.4%. The microarray data were deposited in the NCBI GEO web-based data repository (series ID: GSE9795). Also, 12 samples from LGNs of monkey deprived for four months were hybridized to the HG-U133Aplus2 array, and the microarray data were deposited in the NCBI GEO web-based data repository (series ID: GSE2165).

**Figure 2:**
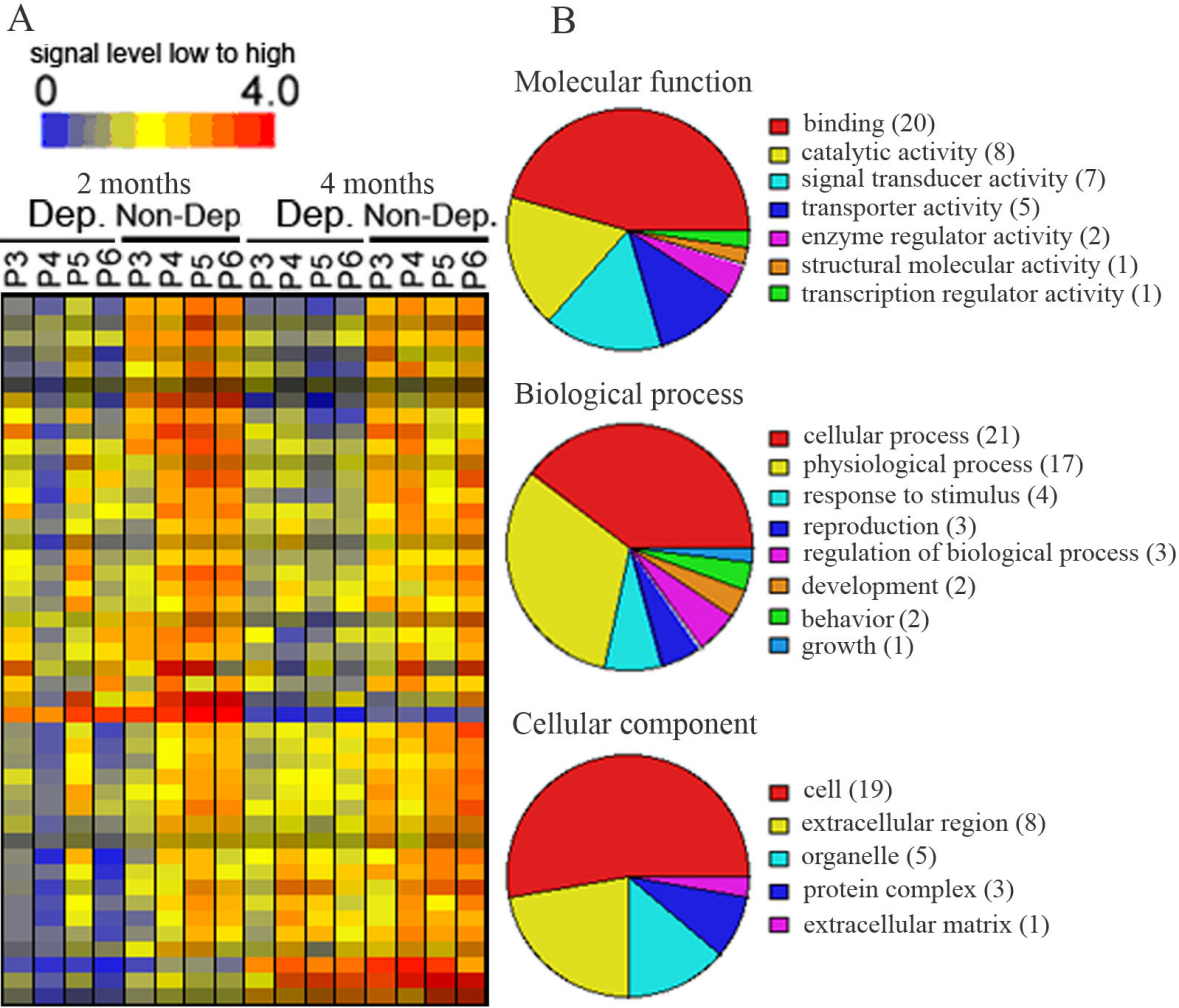
Transcripts that were downregulated in deprived parvocellular layers as compared with non-deprived layers of 2 or 4 months after monocular vision deprivation. **A:** Hierarchical dendogran analysis displayed genes downregulated (blue to green color) in deprived layers as compared with non-deprived layers. **B:** Pie chart displayed functional categorization of genes that were downregulated in deprived layers. Number in parentheses indicated how many genes involved in that category function. P3-P6 represent parvocellular layer III to VI. Abbreviations: Dep: deprived layer; Non-Dep: non-deprived layer; 2 months or 4 months: 2 or 4 months of monocular vision deprivation.

LCM and microarray technology were used to identify genes that were differentially expressed between deprived and nondeprived parvocellular layers of monocular vision-deprived monkeys. Our data showed that, of the 52,699 probe sets, 116 transcripts were differentially expressed between deprived and nondeprived parvocellular layers; 45 genes were downregulated and 71 transcripts were upregulated in deprived layers. The expression levels of 45 downregulated genes were displayed in a hierarchical dendogram generated by the GeneSpring software (Figure 2A), and transcripts were classified according to their functions (Figure 2B). The functions were further classified into molecular functions, biologic processes, and cellular components, which were subclassified according to their activity. Most downregulated genes in the molecular functions were involved in binding (44.4%), in catalytic activity (17.8%), and in signal transducer activity (15.6%). A further analysis of these binding genes revealed that calcium ion binding, nucleic acid binding, and nucleotide binding genes were abundant in this category. The majority of downregulated genes in the biologic process included genes involved in cellular process (46.7%) and physiologic processes (37.8%). Cell-cell signaling and signal transduction genes and genes related to metabolisms represented the major part of the cellular process. The genes downregulated in the cellular components were mainly involved in cell (42.2%), extracellular region (17.8%), and organelle (11.1%). Genes downregulated in deprived parvocellular layers were listed in Appendix 1.

**Figure 3:**
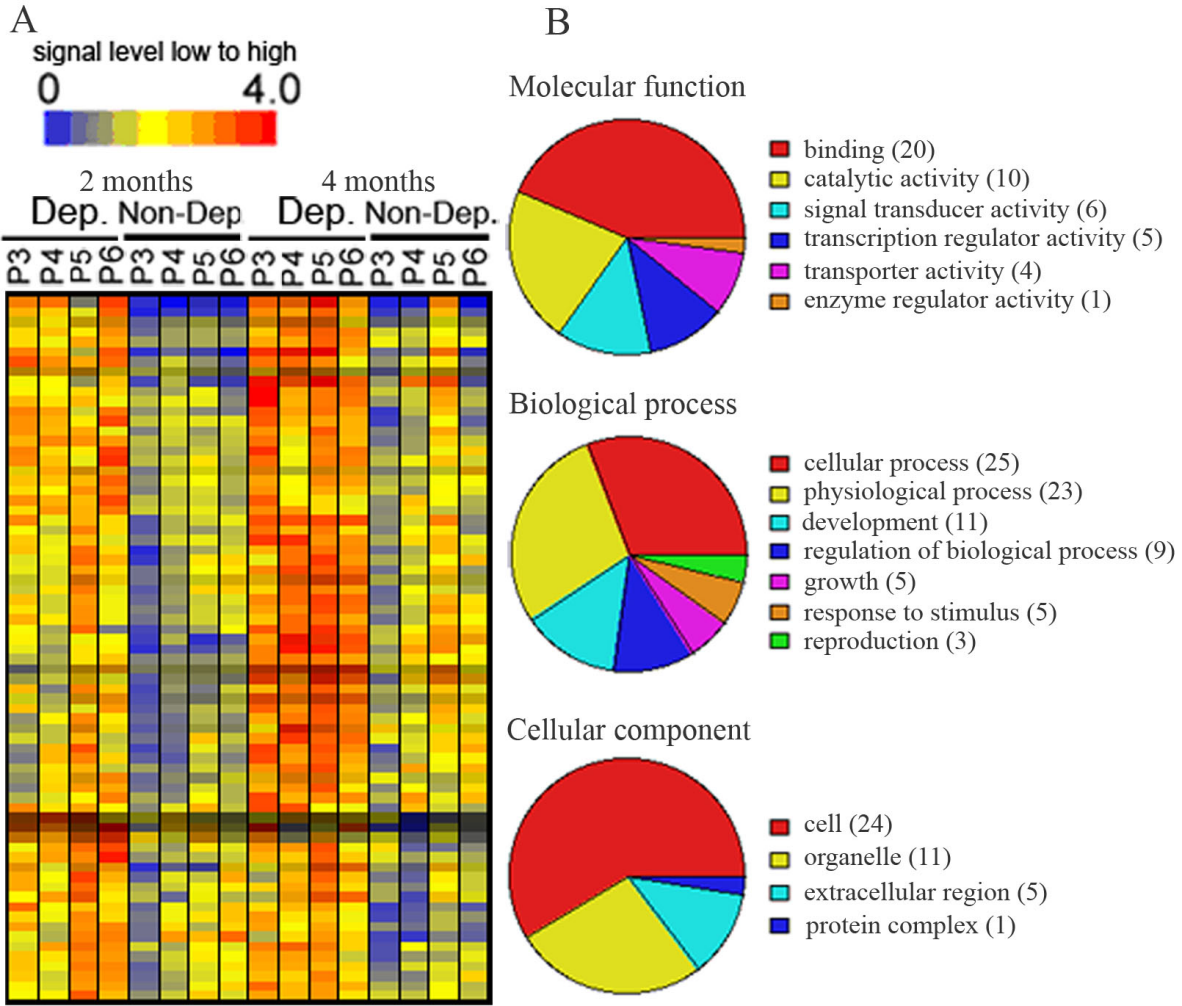
Transcripts that were upregulated in deprived parvocellular layers as compared with non-deprived layers of 2 or 4 months after monocular vision deprivation. **A:** Hierarchical dendogran analysis displayed genes upregulated (orange to red color) in deprived layers as compared with non-deprived layers. **B:** Pie chart displayed functional categorization of genes that were upregulated in deprived layers. Number in parentheses indicated how many genes involved in that category function. P3-P6 represent parvocellular layer III to VI. Abbreviations: Dep: deprived layer; Non-Dep: non-deprived layer.2 months or 4 months: 2 or 4 months of monocular vision deprivation.

The 71 upregulated genes were also displayed in a hierarchical dendogram generated by the GeneSpring software (Figure 3A) and transcripts were classified according to their functions (Figure 3B). In the molecular function, most upregulated genes were involved in binding (28.2%), in catalytic activity (14.1%), and in signal transducer activity (8.4%). A further analysis of these upregulated binding genes revealed that calcium ion binding, nucleic acid binding, and nucleotide binding genes accounted for most of the genes in the binding function category. In the biologic process group, the majority of upregulated genes were involved in cellular process (35.2%), physiologic process (32.4%), development (15.5%), and regulation of biologic process (12.7%). A further analysis showed that cell communication and cell growth/maintenance genes were the major genes involved in the cellular process group. Of these, host-pathogen interaction and signal transduction genes were the major part of the cell communication group. The genes related to cell growth and metabolisms were the major genes in the cell growth/maintenance category. Cell (33.8%) and organelle (15.5%) function genes were heavily represented in upregulated genes in the cellular component group. Genes upregulated in deprived parvocellular layers were listed in Appendix 2.

**Figure 4:**
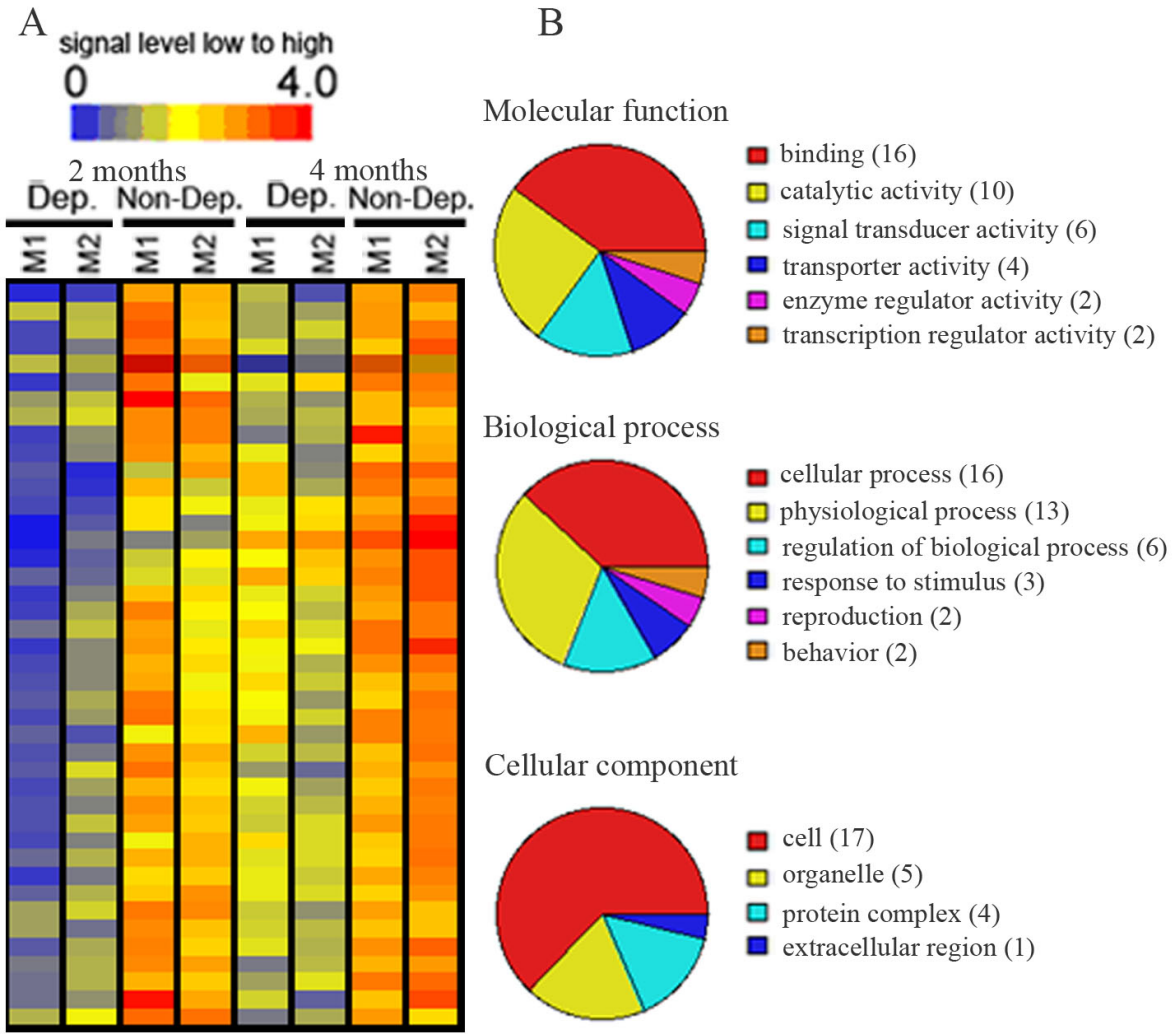
Transcripts that were downregulated in deprived magnocellular layers as compared with non-deprived layers of 2 or 4 months after monocular vision deprivation. **A:** Hierarchical dendogran analysis displayed genes downregulated (blue to green color) in deprived layers as compared with non-deprived layers. **B:** Pie chart displayed functional categorization of genes that were downregulated in deprived layers. Number in parentheses indicated how many genes involved in that category function. M1-M2 represent magnocellular layer I to II. Abbreviations: Dep: deprived layer; Non-Dep: non-deprived layer; 2 months or 4 months: 2 or 4 months of monocular vision deprivation.

**Figure 5:**
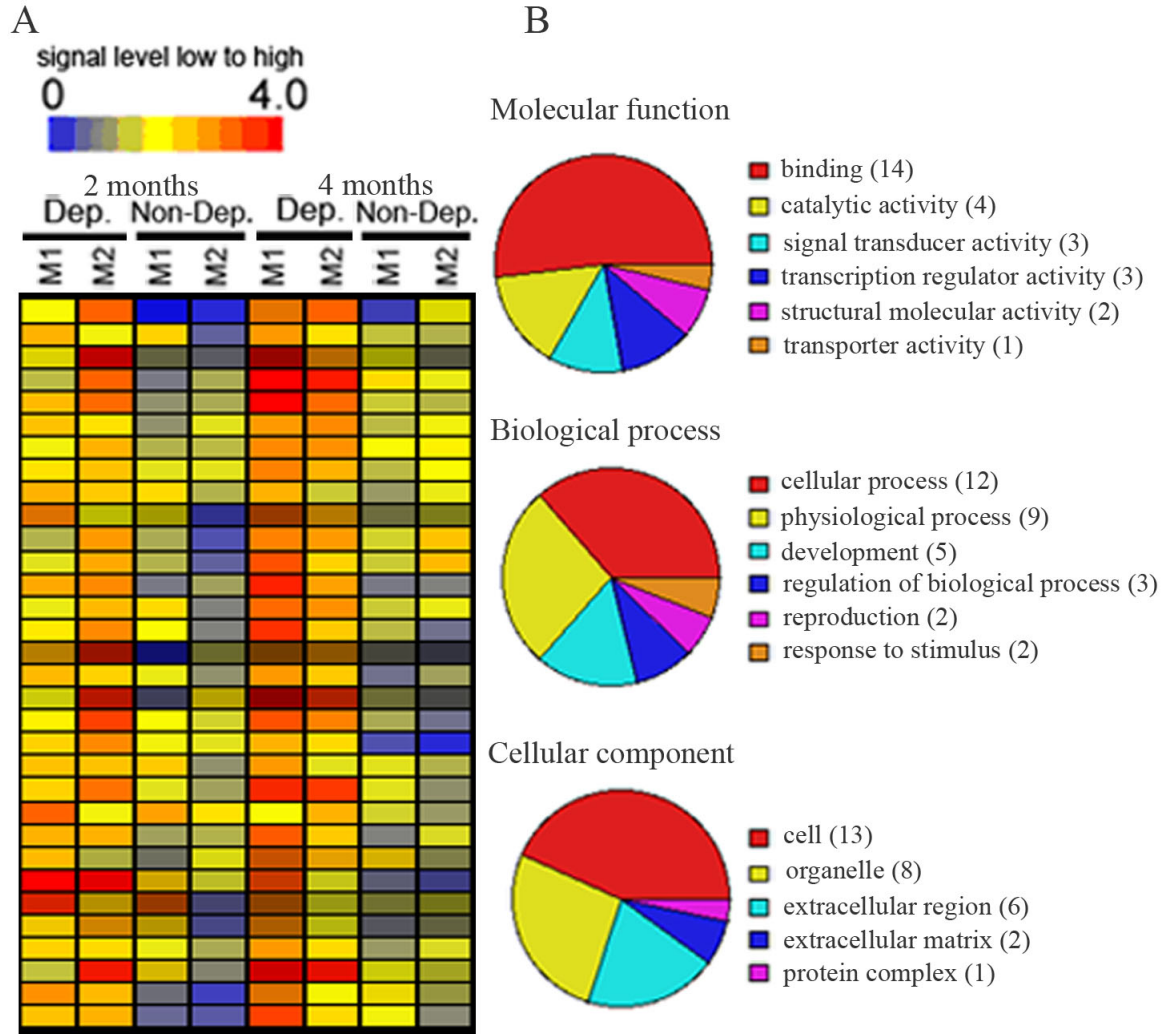
Transcripts that were upregulated in deprived magnocellular layers as compared with non-deprived layers of 2 or 4 months after monocular vision deprivation. **A:** Hierarchical dendogran analysis displayed genes upregulated (orange to red color) in deprived layers as compared with non-deprived layers. **B:** Pie chart displayed functional categorization of genes that were upregulated in deprived layers. Number in parentheses indicated how many genes involved in that category function. M1-M2 represent magnocellular layer I to II. Abbreviations: Dep: deprived layer; Non-Dep: non-deprived layer. 2 months or 4 months: 2 or 4 months of monocular vision deprivation.

There were also 74 transcripts that exhibited different expression levels between the deprived and nondeprived magnocellular layers: 42 genes were downregulated and 32 were upregulated in deprived magnocellular layers. The expression levels of 42 downregulated genes were displayed in a hierarchical dendogram generated by the GeneSpring software (Figure 4A), and transcripts were classified according to their functions (Figure 4B). In the molecular function group, most of the downregulated genes were involved in binding (38.1%), in catalytic activity (23.8%), and in signal transducer activity (14.3%). A further analysis of these downregulated binding genes revealed that calcium ion binding and nucleic acid binding genes were the major part of the binding function category. In the biologic processes group, the majority of downregulated genes were involved in cellular process (38.1%), physiologic process (31%), and regulation of biologic process (14.3%). In the cellular process category, cell-cell signaling and signal transduction genes were the major genes downregulated in the cell communication group, and metabolism related genes were the major genes downregulated in the cell growth/maintenance group. Cell (40.5%), organelle (11.9%), and protein complex (9.5%) genes were heavily represented in the genes downregulated in the cellular component category. Appendix 3 listed downregulated genes in deprived magnocellular layers. The upregulated genes were displayed in a hierarchical dendogram generated by the GeneSpring software (Figure 5A) and transcripts classified according to their functions (Figure 5B). Binding (43.8%) genes was the major genes upregulated in the molecular function category. A further analysis of these upregulated binding genes revealed that nucleic acid binding and nucleotide binding genes were the main genes involved in binding function. In the biologic processes, the majority of upregulated genes were involved in cellular process (37.5%), physiologic process (28.1%), and development (15.6%). Cell-cell signaling, host-pathology interaction, and signal transduction genes played a major part in the cell communication group, and metabolism related genes accounted for the cell growth/maintenance portion in the cellular process category. The genes upregulated in the cellular components group were mainly involved in cell (40.6%), organelle (25%), and extracellular region groups (18.8%). Table 1 listed upregulated genes in deprived magnocellular layers.

**Table 1:** Transcripts that were upregulated in deprived magnocellular layers as compared with non-deprived layers

**Affymatrix ID**	**RefSeq**	**Gene Symbol**	**Transcripts**	**Fold Change**
MmugDNA.2791.1.S1_at	NM_002581	PAPPA	pregnancy-associated plasma protein A, pappalysin 1	9.1
MmuSTS.1876.1.S1_at	NM_080803	COL13A1	collagen, type XIII, alpha 1	8.3
MmuSTS.3434.1.S1_at	NM_170696	ALDH1A2	aldehyde dehydrogenase 1 family, member A2	5.4
MmugDNA.7497.1.S1_at	NM_213609	FAM19A1	family with sequence similarity 19, member A1	4.6
MmugDNA.27876.1.S1_at	NM_002581	PAPPA	pregnancy-associated plasma protein A, pappalysin 1	4.2
MmuSTS.3785.1.S1_at	AY436323	AGT	angiotensinogen (serine/cysteine) proteinase inhibitor	4.2
MmugDNA.18346.1.S1_at	NM_198541	IGFL1	insulin growth factor-like family member 1	3.9
MmugDNA.22116.1.S1_at	NM_006183	NTS	neurotensin	3.8
MmugDNA.13685.1.S1_at			unknown	3.7
MmuSTS.3385.1.S1_at	NM_181523	PIK3R1	phosphoinositide-3-kinase, regulatory subunit 1	3.7
MmugDNA.35490.1.S1_at	NM_006328	RBM14	RNA binding motif protein 14	3.3
MmugDNA.14651.1.S1_at	NM_004904	CREB5	cAMP responsive element binding protein 5	3.2
MmugDNA.37283.1.S1_at	NM_021190	PTBP2	Polypyrimidine tract binding protein 2	3.1
MmugDNA.17471.1.S1_at	NM_024749	FLJ12505	Hypothetical protein FLJ12505	3.0
MmugDNA.38329.1.S1_at	NM_032160	C18orf4	chromosome 18 open reading frame 4	3.0
MmugDNA.37023.1.S1_at	NM_006147	IRF6	interferon regulatory factor 6	2.8
MmugDNA.38004.1.S1_at			Transcribed locus	2.7
MmugDNA.29288.1.S1_at	NM_015393		unknown	2.5
MmugDNA.37083.1.S1_at	NM_145753	PHLDB2	pleckstrin homology-like domain, family B, member 2	2.5
MmugDNA.6639.1.S1_at			Transcribed locus	2.4
MmugDNA.22537.1.S1_s_at	NM_002291	LAMB1	laminin, beta 1	2.2
MmuSTS.3615.1.S1_at	NM_001266	CES1	carboxylesterase 1	2.1
MmugDNA.42270.1.S1_at	NM_013372	GREM1	gremlin 1, cysteine knot superfamily	2.0
MmugDNA.9692.1.S1_at			unknown	2.0
MmugDNA.15114.1.S1_at	NM_001812	CENPC1	centromere protein C 1	1.9
MmugDNA.32812.1.S1_at	NM_006108	SPON1	spondin 1, extracellular matrix protein	1.9
MmugDNA.6252.1.S1_s_at	NM_001670	ARVCF	armadillo repeat gene deletes in velocardiofacial syndrome	1.9
MmuSTS.3940.1.S1_at	NM_007269	STXBP3	syntaxin binding protein 3	1.9
MmugDNA.39862.1.S1_at	NM_001280	CIRBP	cold inducible RNA binding protein	1.8
MmugDNA.8458.1.S1_at	NM_000527	LDLR	low density lipoprotein receptor	1.8
MmugDNA.34471.1.S1_at	NM_025154	UNC84A	unc-84 homolog A (C. elegans)	1.7
MmugDNA.39414.1.S1_at	NM_002130	HMGCS1	3-hydroxy-3-methylglutaryl-Coenzyme A synthase 1	1.7

Affymatrix ID is ID from Affymatrix GeneChip. RefSeq, Gene Symbol, and Transcripts are the gene ID, gene symbol and gene name from GenBank. Number indicated fold change.

### Verification of differential gene expression by quantative reverse transcription polymerase chain reaction

We selected several differentially expressed genes involved in cell-cell signaling and signal transduction as detected by microarray for verification by qRT–PCR. The qRT–PCR results were consistent with the microarray data for all the genes verified. For example, *CRH* and *GABRA1* were downregulated in deprived parvocellular layers by 2.3 fold and 1.7 fold by microarray and 4.3 fold and 2.5 fold by qRT–PCR. Dihydropyrimidinase-like 3 (*DPYSL3*) was upregulated in deprived parvocellular layers by 2 fold by microarray and 2.5 fold by qRT–PCR. The primers used in qRT–PCR and fold changes of genes were listed in Table 2.

**Table 2:** Verification of the data by qRT-PCR

**Gene**	**Primer sequence**	**Microarray fold change**	**qRT-PCR fold change**
NDST4	5’-CCCCAAAGCCAAGATCATCA-3’	-3.7	-3.3
	5’-ACCTCAGAGCAGCTGGATCTTC-3		
CRH	5’-GCAGCAGCAACACAATGTTATTC-3’	-2.3	-4.3
	5’-ACGTTTTCTCACAGGTCTACATTCTC-3’		
KCNC3	5’-CTTGTCACCGCCTGAGACCT-3’	-2.0	-2.7
	5’-GAGATTTGAAGCCCAGTGTCTT-3’		
STAT4	5’-GGTTGTCTGCTCTGCCATTC-3’	-1.9	-2.4
	5’-TTTGGGAATGTCAGGATATAGG-3’		
WNT5A	5’-TTGCAGCGTATCACTGTTATGA-3’	-1.8	-2.5
	5’-TTCAAGTACACTGGGAACAGTTTT-3’		
GABRA1	5’-AAGAGGTCAAGCCCGAAACA-3’	-1.7	-2.5
	5’-AAATAGCAGCGGGAAGGCTAT-3’		
NQO2	5’-CCACGAAGCCTACAAGCAAAG-3’	-1.7	-3.1
	5’-AGTACAGCGGGAACTGAAATATCAC-3’		
CAMK2A	5’-ATCACTGGCCTCTGTCCTTG-3’	1.8	2.3
	5’-ATGGGATCACTGGGCCTTACT-3’		
CREB5	5’-GCTCACCACTCACAGAACAGAC-3’	1.9	2.6
	5’-TTAAAAGAAAGGACGCATGGTA-3’		
DPYSL3	5’-GCTGACACCTGAGCCTGGAT-3’	2.0	2.5
	5’-GGAGAAAGCCTGGGAAGCTT-3’		
PIK3R1	5’-TCATTGAACAGCAAAGTAGGATTCA-3’	2.4	3.2
	5’-CGGTGGGCAAGCTACACTGTA-3’		
SCN3B	5’-GACCATAGCTGCTTCCTTTTCT-3’	2.4	3.7
	5’-AGAAGCAGTGAGTGGGATTAGG-3’		
HRK	5’-GGAGCCCAGAGCTTGAAAGG-3’	4.1	4.4
	5’-CCCCAGTCCCATTCTGTGTT-3’		
ALDH1A2	5’-AGTGTCTTCTGCAATGCAAGC-3’	5.4	6.3
	5’-CATTTCTCTCCCATTTCCAGAC-3’		
CRH	5’-CTCACAGCAACAGGAAACTC-3’	-2.3	primers for in situ
	5'-CTCTTACACAACCAAACTGACCAA-3'		
GABRA1	5’-TATTGCCGTGTGCTATGCCTTTGT-3’	-1.7	primers for in situ
	5’-AGATGGGAATTACTGCGTTGAGAA-3’		

The PCR primers were designed based on human gene sequences derived from GenBank. Primers for in situ hybridization probes were generated by PCR reactions using monkey corticotrophin-releasing hormone (*CRH*; accession number NM_000756) and gamma-aminobutyric acid A receptor, alpha 1 (*GABRA1* from accession number NM_000806).

### Verification of differential gene expression by in situ hybridization

**Figure 6:**
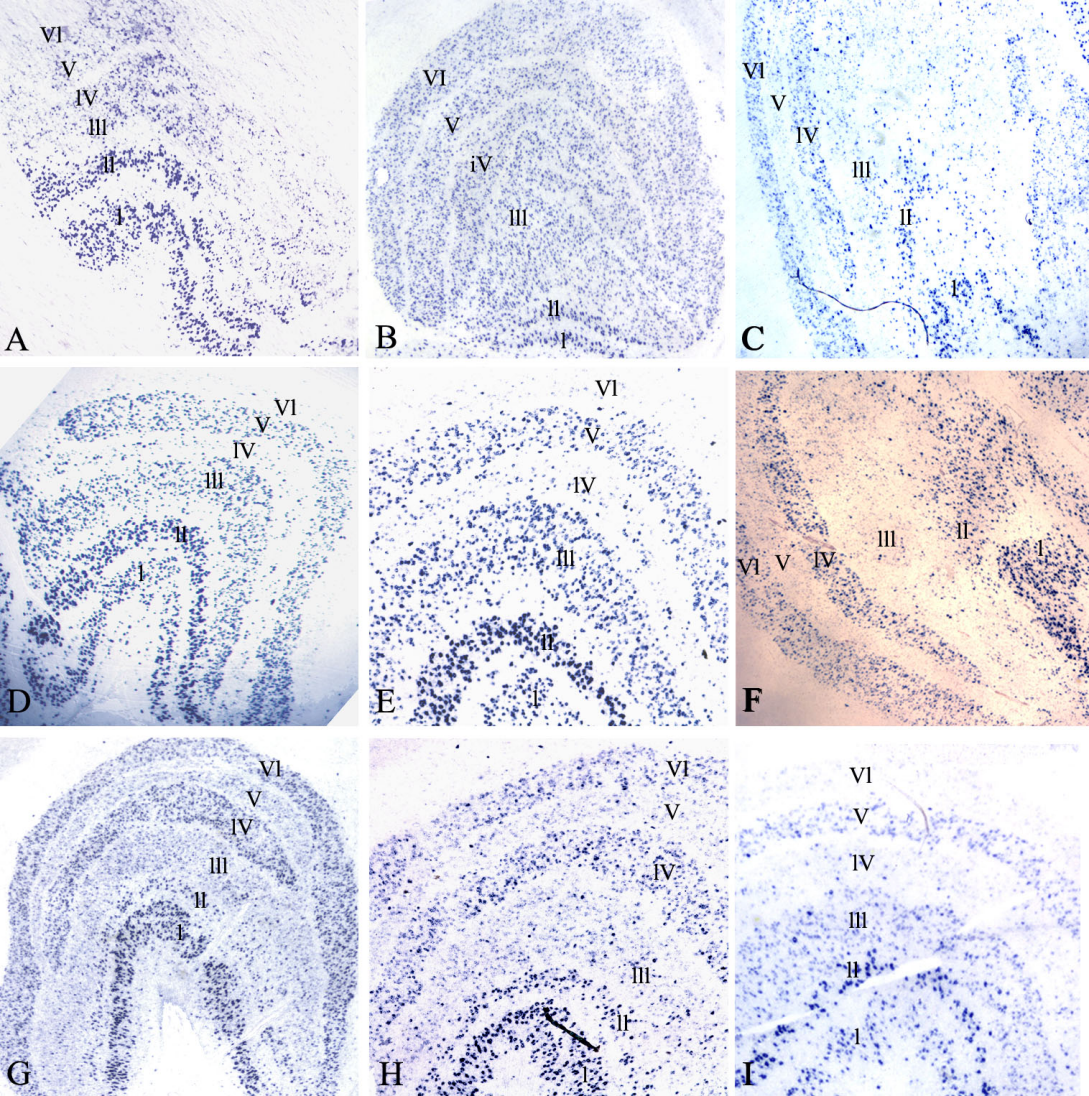
In situ hybridization of CRH in control and LGN. **A** and **B**: CRH expression in the LGN of two control monkeys (C1and C2). **C:** Ipsilateral LGN of a monkey that was monocular vision-deprived for four months (MD5). **D:** CRH showed decreased expression in deprived layers (layers I, IV, and VI) in the LGN contralateral to the eye from a monkey that was monocular vision-deprived for four months (MD3). **E.** Higher-magnification photomicrograph of **D**. **F:** Ipsilateral LGN from a monkey that was monocular vision-deprived for two (MD2). **G.** CRH showed decreased expression in deprived layers (layers II, III, and V) in the LGN ipsilateral to the eye from a monkey that was monocular vision-deprived for four months (MD4). **H:** Higher-magnification photomicrograph of **G**. **I:** Contralateral LGN eye from a monkey that was monocular vision-deprived for four months (MD4).

**Figure 7:**
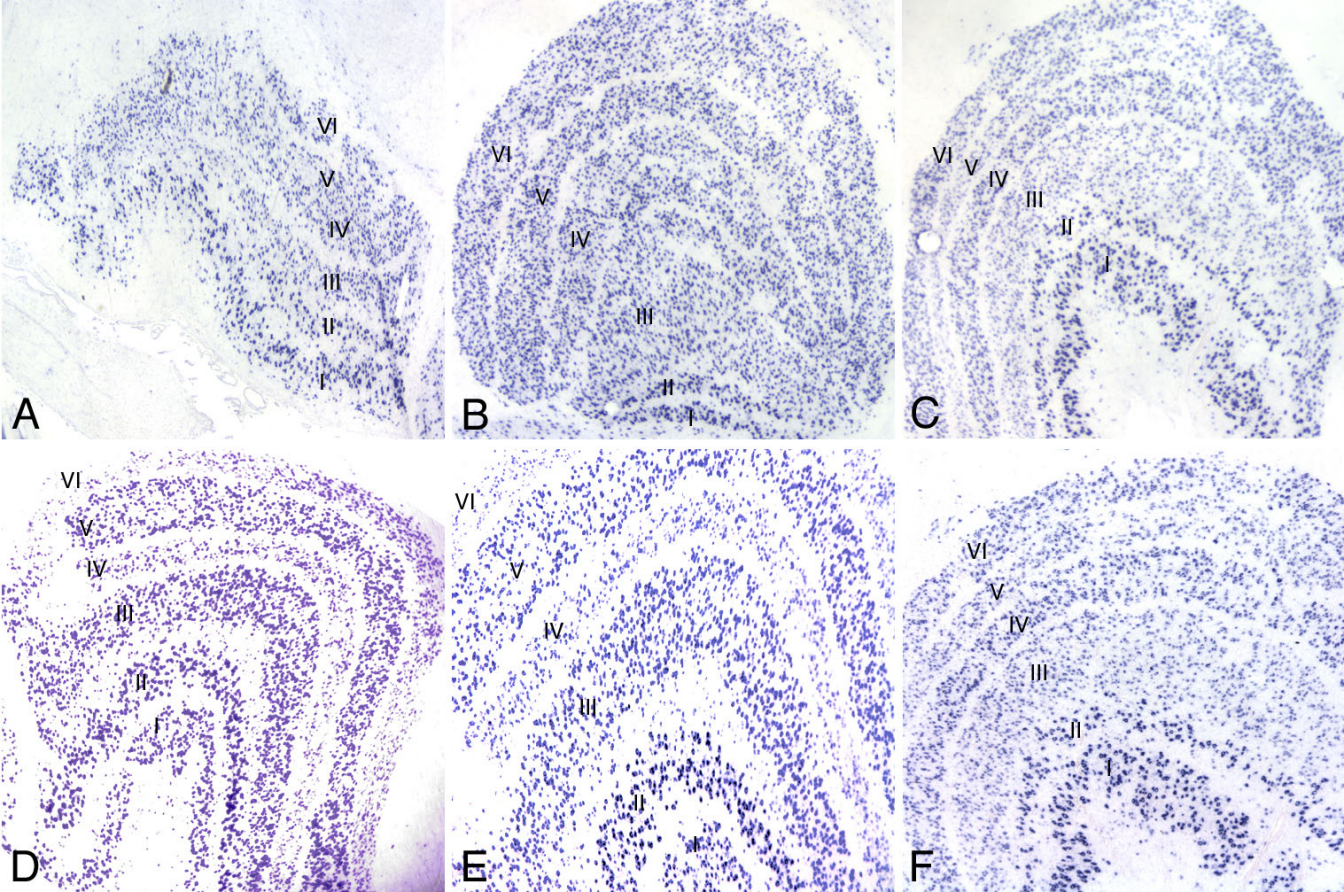
In situ hybridization of GABRA1 in control and deprived LGN. **A** and **B:** GABRA1 expression in the LGN of two control monkeys (C1 and C2). **C:** GABRA1 showed decreased expression in deprived layers (layers II, III, and V) in the LGN ipsilateral to the eye from a monkey that was monocular vision-deprived for four months (MD4). **D:** GABRA1 showed decreased expression in deprived layers (layers I, IV, and VI) in the LGN contralateral to the eye from a monkey that was monocular vision-deprived for four months (MD3). **E:** Higher-magnification photomicrograph of **D**. **F:** Higher-magnification photomicrograph of **C**.

**Figure 8:**
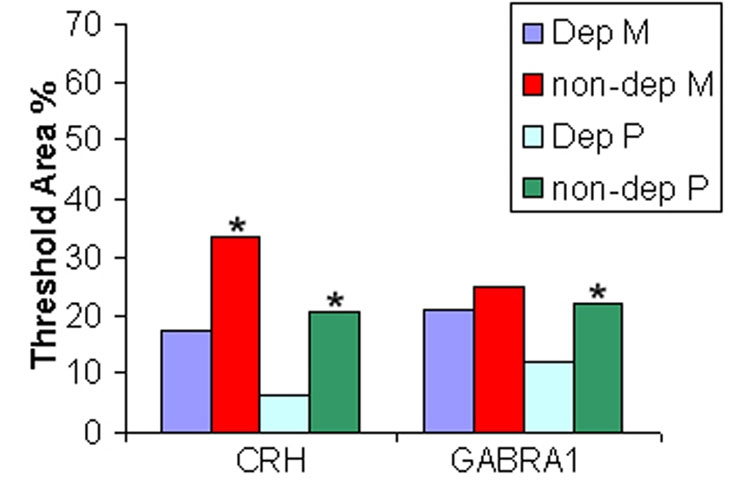
Quantification for in situ hybridization of CRH and GABRA1 in deprived LGN. Percent threshold area represents the percentage of area in a given layer that contains a positive in situ hybridization signal. The purple colored graph bar is Dep M which indicates deprived magnocellular layers. The red colored graph bar is non-dep M which indicates nondeprived magnocellular layers. The light blue colored graph bar is Dep P which indicates deprived parvocellular layers. The green colored graph bar is non-dep P which indicates nondeprived parvocellular layers. The asterisk Denotes a p<0.05 for deprived layers compared to nondeprived layers.

Several differentially expressed genes involved in cell-cell signaling as detected by microarray were analyzed by in situ hybridization. Primers generated for in situ probes are listed in Table 2. Expression of *CRH* and *GABRA1* was downregulated in deprived layers of deprived monkeys. The microarray results were confirmed by in situ hybridization. The density of staining was markedly reduced in deprived layers II, III, and V (Figure 6C,F,G,H) and I, IV, and VI (Figure 6D,E,I) for *CRH* (Figure 6). The density of staining was markedly reduced in deprived layers II, III, and V (Figure 7C,F) and I, IV, and VI (Figure 7D,E) for *GABRA1* (Figure 7). The density of staining of *CRH* and *GABRA1* was quantified for LGN in deprived monkeys (Figure 8). In normal control LGN, the density of staining for *CRH* and *GABRA1* was evenly distributed in parvocellular or magnocellular layers. These findings support the reliability of our microarray results.

## Discussion	

Understanding the molecular pathogenesis of amblyopia is a necessary step toward the discovery of effective therapies. Our present study demonstrates that we have established a powerful model to study the molecular mechanisms related to monocular vision-deprivation-induced amblyopia in nonhuman primates. By using laser capture microdissection and DNA microarray techniques in a monocular vision-deprived monkey, we have obtained a snapshot of gene expression in LGN at a time when anatomic and cellular alterations resulting from visual deprivation are well established.

Distinct anatomic and cellular alterations have been shown to occur in the LGN of monkeys that have undergone two or four months of visual deprivation. Our microarray analysis revealed 190 genes with altered expression due to visual deprivation in the parvocellular and magnocellular layers of LGN. Although this is a relatively small number, the genes identified are involved in many diverse processes and are quite likely to play roles in the pathogenesis of amblyopia associated with monocular vision deprivation.

The primary anatomic changes of the deprived LGN parvocellular layer include neuronal loss and atrophy. Consistent with these findings, our expression profiling discovered several differentially expressed genes that were reported to be involved in controlling neuronal apoptosis and atrophy, including *CRH* which might prove to be particularly important in driving the anatomic changes associated with monocular vision deprivation. Reduced *CRH* expression was detected in the deprived layers of LGN in monkeys deprived for two or four months (cross-species hybridization using the human chip showed the same result, which was deposited in the GEO as GSE2165). Once thought to be only involved in maintaining neuroendocrine responses to induce cell-cell signaling and signal transduction, the role of CRH has now been expanded and is thought to be involved in several apoptotic pathways. In primary neuronal culture, CRH was protective against cell death caused by an amyloid-beta peptide. This effect was blocked by a CRH receptor antagonist and by an inhibitor of cyclic AMP-dependent protein kinase. CRH can also protect neurons against cell death caused by lipid peroxidation and the excitotoxic neurotransmitter glutamate [27]. Therefore, the reduction in CRH expression levels observed in the present study suggests that CRH might account for the reduction of LGN neuron number in the deprived layers. CRH stimulates cAMP production in various regions of the central nervous system and binds specifically to CRH receptor type 1 (which also was downregulated in our study), which leads to stimulation of multiple G-proteins associated with several putative intracellular signaling pathways [28]. Downregulation of CRH expression has also been observed in some neurodegenerative diseases such as Alzheimer disease [29-31] and Huntington disease [32]. A potential role for reduced CRH in neuron loss in the LGN of deprived monkeys has not been previously considered, but could be of particular importance.

GABA, the major inhibitory neurotransmitter in the mammalian brain, functions through the GABA-A receptor (GABRA). GABRA is a ligand gated chloride channel that has been shown to be involved in visual cortical plasticity. The onset of critical period can be delayed by low levels of GABA and accelerated by enhancing its inhibition [33]. One subunit of this receptor is GABRA alpha 1 (GABRA1). Our study showed a reduction of *GABRA1* mRNA in deprived layers of LGN (cross-species hybridization using the human chip showed the same result, which was deposited in the GEO as GSE2165). Our finding further supports the importance of GABA activity in the pathology of monocular vision deprivation. The finding is also consistent with previous studies on monkeys [34,35], which showed reduced GABA activity in the deprived LGN. Hendry and Miller [36] reported decreased GABRA1 protein, and Huntsman and colleagues [37] reported decreased mRNA in deprived LGN.

Prior gene expression profiling studies to compare deprived and nondeprived layers of the LGN in monocular vision-deprived monkey are scarce. This is the first paper to study deprived and nondeprived layers of the LGN in the monocular vision-deprived monkey at the molecular level by using LCM and Rhesus chip microarray. Lachance and Chaudhuri [21] used similar techniques on the normal vervet monkey (an Old World species) and samples cross-hybridized to the Rhesus array. Prasad et al. [38,39] used monkey cDNA array to study gene profiling of parvocellular and magnocellular layers, but did not dissect individual layers. In the present study, we used laser capture microdissection, which allowed a more precise dissection of each layer. We also used genome-wide expression profiling technology to allow screening of more genes. Our microarray data proved reliable as several selected differentially expressed genes were verified by qRT–PCR and in situ hybridization.

In summary, by using laser capture microdissection and DNA microarray techniques in a monocular vision-deprived amblyopia monkey model, we found significant differences in the gene expression profile between deprived and nondeprived parvocellular layers and magnocellular laminae. We identified genes that were upregulated or downregulated in LGN associated with visual deprivation. These alterations in gene expression may play a critical role in the molecular pathogenesis of amblyopia. The genes identified in this study are involved in many diverse processes, including cell-cell signaling/binding (calcium ion binding, nucleic acid and nucleotide binding), catalytic activity/metabolisms, and signal transducer activity. Change of expression levels of these genes may indeed drive the anatomic and physiologic alterations from visual deprivation. The findings may provide insights into understanding molecular pathogenesis of amblyopia. It is also possible that some anatomic and physiologic alterations seen in visual deprivation may be caused by post-transcriptional regulation of genes. Further identification of molecular pathophysiologic pathways of monocular vision deprivation and gene expression changes in the visual cortex will increase our understanding of disease causing mechanisms. These may eventually lead to effective therapeutic interventions to slow or reverse the consequences of monocular vision deprivation during visual development, such as amblyopia from congenital cataract. We focused on the genes with similar expression changes after two and four months of monocular vision deprivation. However, it is possible that changes of genes at one time point related to amblyopia and may or may not related to maturation might be missed by the current selection of differentially expressed genes. However, all of our monkeys displayed profound amblyopia at both two months and four months time points, indicating that a steady-state of amblyopia has been reached at these two time points. The model applied here would be also useful in designing future studies to unravel the molecular alterations in other parts of the visual pathways including the primary visual cortex.

## Appendix 1. Transcripts that were downregulated in deprived parvocellular layers as compared with non-deprived layers.

To access the data, click or select the words “Appendix 1”. This will initiate the download of a (pdf) archive that contains the file. Affymatrix ID is ID from Affymatrix GeneChip. RefSeq, Gene Symbol, and Transcripts are the gene ID, gene symbol and gene name from GenBank. Number indicated fold change, negative indicated downregulated. A positive fold change means the corresponding gene was upregulated in deprived layers.

## Appendix 2. Transcripts that were upregulated in deprived parvocellular layers as compared with non-deprived layers.

To access the data, click or select the words “Appendix 2”. This will initiate the download of a (pdf) archive that contains the file. Affymatrix ID is ID from Affymatrix GeneChip. RefSeq, Gene Symbol, and Transcripts are the gene ID, gene symbol and gene name from GenBank. Number indicated fold change.

## Appendix 3. Transcripts that were downregulated in deprived magnocellular layers as compared with non-deprived layers

To access the data, click or select the words “Appendix 3”. This will initiate the download of a (pdf) archive that contains the file. Affymatrix ID is ID from Affymatrix GeneChip. RefSeq, Gene Symbol, and Transcripts are the gene ID, gene symbol and gene name from GenBank. Number indicated fold change, negative indicated downregulated.
